# Genetically similar strains of *Escherichia coli* O157:H7 isolated from sheep, cattle and human patients

**DOI:** 10.1186/1746-6148-8-200

**Published:** 2012-10-24

**Authors:** Robert Söderlund, Ingela Hedenström, Anna Nilsson, Erik Eriksson, Anna Aspán

**Affiliations:** 1 Department of Bacteriology, National Veterinary Institute, Uppsala, 75189, Sweden; 2 Swedish Institute for Communicable Disease Control, Solna, 17182, Sweden; 3 Department of Clinical Sciences, Swedish University of Agricultural Sciences, Uppsala, 75651, Sweden; 4 Department of Animal Breeding and Genetics, Swedish University of Agricultural Sciences, Uppsala, 75651, Sweden

**Keywords:** O157:H7, VTEC, STEC, Sheep, MLVA, PFGE, Clade 8

## Abstract

**Background:**

Comparatively little is known about the prevalence or the molecular characteristics of the zoonotic pathogen *E. coli* O157:H7 in the sheep reservoir. To investigate this and determine the host specificity of subclones of the bacterium, we have conducted a slaughterhouse prevalence study in sheep and compared the collected isolates to O157:H7 previously isolated from cattle and human patients.

**Results:**

Verotoxin-producing O157:H7 was found in 11/597 (1.8%) of samples from sheep in Swedish slaughterhouses, 9/492 faecal (1.8%) and 2/105 ear samples (1.9%). All positive sheep were < 6 months old. Pulsed field gel electrophoresis typing revealed exact matches between isolates from the sheep prevalence study and human patients as well as between isolates from sheep and cattle. In one case, matching isolates were found in sheep, cattle, and a human patient in the same municipality. Identical PFGE profiles generally corresponded to similar but non-identical multi-locus VNTR profiles. In one sheep sample, SNP-typing found the highly virulent clade 8 variant of O157:H7. The virulence gene profiles of sheep isolates from the prevalence study and three sheep farms linked to cases of human illness were investigated by PCR detection (*eaeA*, *hlyA*, *cdtV-B*, *vtx*_*1*_), and partial sequencing of *vtx*_*2*_. The observed profiles were similar to those of cattle strains investigated previously.

**Conclusions:**

The same pathogenic subtypes of VTEC O157:H7, including the highly virulent clade 8, appear to be present in both sheep and cattle in Sweden, suggesting strains can circulate freely between ruminant reservoirs.

## Background

Verotoxin-producing *Escherichia coli* (VTEC) O157:H7 is a zoonotic pathogen spread by ruminants which are asymptomatic carriers of the bacterium. Strains capable of causing haemorrhagic colitis in humans are referred to as enterohaemorrhagic *E. coli* (EHEC). The main source of human infection is cattle, but sheep are also considered a significant reservoir [1]. A study in the UK found O157:H7 contamination to be more common in raw lamb meat products compared to beef [2]. The sheep’s fleece is a potential source of bacterial contamination for the carcass at slaughter, particularly if the wool is long [3]. Dairy products such as cheese based on unpasteurized milk and environmental contamination represent other likely routes of transmission from sheep to humans [1].

Unlike in cattle, relatively little is known about the genotypic characteristics of O157:H7 found in sheep. Recent studies in European countries have found strains with identical virulence gene profiles and phage types in bovine and ovine hosts, but with no shared pulsed-field gel electrophoresis (PFGE) profiles between species [4,5]. There is strong evidence that certain genetic groups of O157:H7 are over-represented in isolates linked to human cases of disease compared to their prevalence in cattle [6,7]. The presence or absence of such groups in sheep is therefore of importance for risk assessment and epidemiological tracing. In collaboration between the Swedish National Veterinary Institute and the Swedish Institute for Communicable Disease Control, we have used molecular typing methods to compare isolates from a prevalence study performed on sheep to isolates from human patients and isolates from prevalence studies performed on cattle. We have also analysed the genotypic characteristics of strains of O157:H7 isolated from sheep farms linked to cases of human disease. The aim was to characterize VTEC O157:H7 strains isolated from sheep and to determine if the same genotypes occur in sheep as in cattle and human patients.

## Results and discussion

Verotoxin-producing O157:H7 was found in 11/597 (1.8%) of analyzed samples from sheep in Swedish slaughterhouses, 9/492 faecal (1.8%) and 2/105 ear samples (1.9%). The bacterium was only found in animals less than six months old and was geographically restricted to southern Sweden. While both of these observations are in agreement with data from Swedish cattle [8], the differences were not significant in sheep due to the low number of sampled animals > 6 months old and the low number of sampled animals in slaughterhouses in the northern part of the country (see Additional file 1 for details). Compared to what has previously been found in studies on cattle [9], the prevalence of VTEC O157:H7 in Swedish sheep appears to be low although this comparison is based on only eleven positive sheep samples altogether. While O157:H7 tends to be more frequently isolated from ear samples than faecal samples from cattle [9], no such difference was observed in sheep. Previous studies in different European countries have reported prevalence estimates in sheep between 0.2% and 8.7% [1], although comparison is complicated by differences in methodology between studies.

All isolates carried genes encoding intimin (*eaeA*) and haemolysin (*hlyA*). Three isolates carried cytolethal distending toxin (*cdtV-B*), and had multi-locus variable number tandem repeat analysis (MLVA) profiles identifying them as closely related to a *cdt*-positive genetic group previously described as common and geographically widely distributed among Swedish cattle [10]. The majority of the isolates had only the verotoxin variant 2c, while a few had combinations (2+2c, 1+2c). Single nucleotide polymorphism (SNP) typing revealed a single clade 8 isolate, which carried the verotoxin variants 2 + 2c. Clade 8 is a genetic subgroup which has caused several large outbreaks in North America and been shown to cause more severe disease compared to other types of O157:H7 [6]. Compared to the overall prevalence in cattle this genotype is highly over-represented in Swedish cattle farms linked to cases of human O157:H7 infection [7]. Most sheep isolates either belonged to lineage I/II (LSPA-6 211111) [11], or differed from this profile only by 2 bp in *yhcG* (213111). Detailed results from the molecular subtyping are presented in Table 1.

**Table 1:** Virulence genes, PFGE and MLVA profiles of VTEC O157:H7 isolated from Swedish sheep

**PFGE type**	**Source**^**1**^	**Year**	***vtx*-genes**^**2**^	***eaeA***	***hlyA***	***cdtV-B***	**Clade 8**^**3**^	**LSPA-6**	**MLVA profile**^**4**^	**Exact matches**
K	PS / M	2007	1,2c	+	+	-	-	213111	15-8-14-3-9-7-5-10	PFGE: Human 2008
Va	PS / I	2008	1	+	+	-	-	213111	NA-7-16-5-9-6-5-11	
Vb	PS / I	2007	1, 2c^INS^	+	+	-	-	213111	NA-7-15-5-9-6-5-11	
La	PS / E	2008	2c	+	+	-	-	211111	5-7-14-4-6-6-NA-5	
Lb	PS / E	2008	2c	+	+	-	-	211111	4-7-NA-5-3-9-5-8	
Lc	PS / E	2008	2,2c	+	+	-	+	211111	17-7-20-4-4-7-8-5	
Ld	PS / E	2008	2c	+	+	-	-	223323	14-7-11-3-4-5-9-6	PFGE: Human 2003, Cattle 2005 (x7)
Ld	PS / E	2008	2c	+	+	-	-	223323	14-7-11-3-4-5-9-6	PFGE: Human 2003, Cattle 2005 (x7)
Sa	PS / R	2008	2c	+	+	+	-	211111	5-7-12-4-6-6-7-5	
Sb	PS / R	2008	2c	+	+	+	-	211111	5-7-13-4-9-6-7-5	
Sb	PS / R	2008	2c	+	+	+	-	211111	5-7-13-4-9-6-7-5	
Y	H / M	2004	2c	+	+	+	-	211111	5-7-14-4-6-6-6-5	MLVA: Cattle 2008 (x2)
T	H / M	2006	1,2c	+	+	-	-	213111	NA-7-10-3-8-7-5-7	
E	H / M	2009	2c	+	+	-	-	223323	22-8-11-3-4-5-6-6	

^1^Sources are specified as the prevalence study (PS) or farms linked to human cases of illness (H), and by county, where M = Halland, I = Gotland, E = Östergötland and R = Skaraborg.

^2^2c = Stx2c-O157-FLY16; 2 = Stx2-O157-EDL933. ^INS^ = IS1209v-like insert in the gene.

^3^Clade 8 status of isolates assessed by SNP analysis for SNP539.

^4^NA = no amplicon for this locus.

PFGE typing found an exact match between a sheep isolate from 2007 (PFGE type K) and an isolate from a human patient in 2008. These isolates originated from the same part of the country, approximately 100 km apart. They differed at only one of eight VNTR loci in the MLVA protocol. A sheep isolate (PFGE type Ld) from a slaughterhouse in Linköping was an exact PFGE match to a human patient isolate from the same town. The isolate also exactly matched three cattle isolates from samples taken in the same slaughterhouse, as well as four cattle isolates from a second slaughterhouse. The three of these seven cattle isolates that were from the slaughterhouse in Linköping, the sheep isolate, and the human isolate had moderately similar MLVA-profiles. There were four identical loci shared by the sheep and human isolates and four to five identical loci shared by the sheep and cattle isolates. These observations of exact PFGE matches corresponding to similar but non-identical MLVA profiles supports previous conclusions [7] that MLVA profiles can be more discriminatory and presumably change more rapidly. Full PFGE and MLVA typing data is presented in Additional file 2. The MLVA profile differences, as well as the fact that the isolates were found in the different host species over a period of five years, suggests that the sheep farms in question were not directly involved with the cases of human disease. Rather, it seems that clones of O157:H7 circulate among both sheep and cattle farms in a region with occasional transmission to humans from either of the reservoirs. Both the sheep isolates matched to human isolates belonged to groups that appear to cause human disease more rarely or with less severe symptoms compared to clade 8. One of them has a LSPA-6 profile suggesting it belongs to lineage II, which has been reported to occur more frequently in cattle compared to patients [12]. MLVA matching produced no exact match between sheep and human isolates. There was a single exact MLVA match between sheep (PFGE type Y) and two cattle isolates from a different part of the country. Sheep isolates did not form any distinct clusters, by either typing method, compared to the cattle isolates. In general, the sheep isolates found in this study had characteristics very similar to the cattle isolates available for comparison, and the typing results did not support the existence of any particular subgroup of O157:H7 that preferentially colonized sheep. However, due to the low prevalence of O157:H7 in this study, these conclusions are based on very few sheep isolates compared to the extensive data available from Swedish cattle and patients.

## Conclusions

Highly similar genotypes of VTEC O157:H7 are found in both cattle and sheep and cause human illness in Sweden. This suggests that pathogenic strains can circulate freely between both ruminant reservoirs. Furthermore, the highly virulent clade 8 can be found in sheep as well as cattle. This information will be of importance for future efforts to trace sources of infection and reduce the burden of disease caused by O157:H7.

## Methods

A total of 492 faecal samples and 105 ear samples from sheep were collected at nine slaughterhouses in Sweden during a period of one year starting in October 2007. The number of samples collected each month and at each slaughterhouse was proportional to the number of sheep slaughtered. Of the sampled animals, 507 (85%) were < 6 months old. *E. coli* O157 was isolated from the samples as previously described [9], except that ear samples were collected by cutting the ear at the base and dividing it in two halves lengthwise, one of which was used for isolation. Additionally, representative isolates from each of three Swedish sheep farms where O157:H7 had infected humans in 2004, 2006 and 2009 were included. These sheep isolates had previously been matched by PFGE to isolates from patients with connections to the farm. MLVA; SNP-typing (for SNP 539 only) to identify clade 8 isolates; PCR detection of *vtx*_*1*_*, vtx*_*2*_, *eaeA*, *hlyA, fliC;* and partial sequencing to determine *vtx*_*2*_ subtype were all performed as described previously [7]. MLVA data was calibrated against sequenced reference isolates to remove systematic errors. PFGE was performed on all sheep isolates by both institutes separately using the enzyme XbaI and matched against local databases to ensure full compatibility with existing data. For human isolates, the PulseNet PFGE protocol [13] was used, cattle isolates were analyzed as previously described [9]. PCR detection of the *cdtV-B* gene was performed according to a published method [14]. LSPA-6 was performed according to the original protocol [12] but with all forward primers FAM-labeled and PCR products analyzed using capillary gel electrophoresis in the same way as for the MLVA assay. EDL933 was used for size correction (profile 111111). Novel alleles were verified by sequencing, allele “3” for *yhcG* is 392 bp long, allele “3” for *rbsB* is 214 bp.

For comparison, PFGE data were available for 116 cattle isolates from a prevalence study performed on Swedish cattle in 2005-2006 [9] and for 850 isolates from Swedish patients in 2001-2009. MLVA data were available for matching from 382 cattle prevalence study isolates between 1996 and 2009 [9,15] and from 110 human patient isolates isolated in 2008-2009.

## Abbreviations

EHEC: Enterohaemorrhagic *Escherichia coli*; LSPA-6: Lineage-specific polymorphism assay-6; MLVA: Multi-locus variable number tandem repeat analysis; PCR: Polymerase chain reaction; PFGE: Pulsed field gel electrophoresis; SNP: Single nucleotide polymorphism; VNTR: Variable number tandem repeat; VTEC: Verotoxin-producing *Escherichia coli*.

## Competing interests

The authors declare no competing interests.

## Authors' contributions

AA and EE conceived and designed the study. RS, IH and AN planned and performed the molecular analysis of bacterial isolates. All authors contributed to the analysis and interpretation of data. All authors read and approved the final manuscript.

## Supplementary Material

Additional file 1 Age distribution of sampled animals and geographic locations of slaughterhouses included in the study. Click here for file

Additional file 2 Clustering and comparison of isolates based on PFGE. Click here for file
