# Supplementary material for: Genetically similar strains of Escherichia coli O157:H7 isolated from sheep, cattle and human patients
Source: BMC Vet Res. 2012 Oct 24;8:200. doi: 10.1186/1746-6148-8-200 (PMC3514354; doi:10.1186/1746-6148-8-200)
Supplement: Additional file 1 — Age distribution of sampled animals and geographic locations of slaughterhouses included in the study. [file 1746-6148-8-200-S1.pdf]

Supplementary file 1

Age distribution of sampled animals and geographic locations of slaughterhouses included in the study

Slaughterhouse location	Tot. samples taken / no. positive	< 6 months old / no. positive	> 6 months old / no. positive
Skellefteå (Ske)	16 / 0	14 / 0	2 / 0
Ullånger (Ull)	14 / 0	12 / 0	2 / 0
Ickholmen (I)	6 / 0	6 / 0	0 / 0
Bäsinge (B)	28 / 0	21 / 0	7 / 0
Uppsala (Upp)	35 / 0	33 / 0	2 / 0
Linköping (L)	198 / 5	170 / 5	28 / 0
Skara (S)	158 / 3	127 / 3	31 / 0
Visby (V)	89 / 2	80 / 2	9 / 0
Kävlinge (K)	53 / 1	44 / 1	9 / 0
**Total**	597 / 11	507 / 11	90 / 0

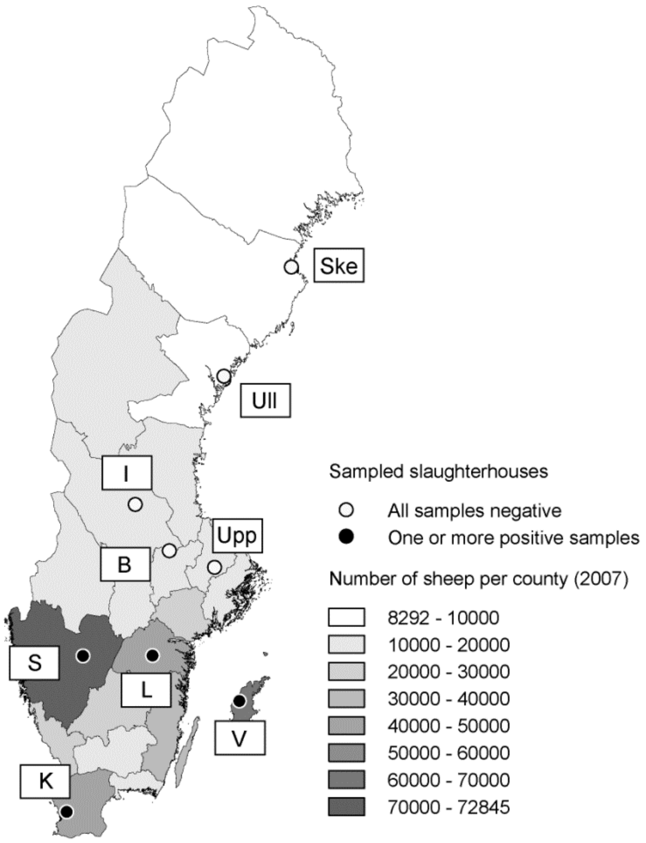

Map created in Quantum GIS 1.7.3 with administrative boundaries at county level from the GADM database (www.gadm.org), version 2.0. For comparison, total number of sheep per county, data from Yearbook of Agricultural Statistics 2008, Swedish Board of Agriculture. (http://www.sjv.se/amnesomraden/statistik/ja.4.7502f61001ea08a0c7fff104195.html)
