# Supplementary material for: Genetically similar strains of Escherichia coli O157:H7 isolated from sheep, cattle and human patients
Source: BMC Vet Res. 2012 Oct 24;8:200. doi: 10.1186/1746-6148-8-200 (PMC3514354; doi:10.1186/1746-6148-8-200)
Supplement: Additional file 2 — Clustering and comparison of isolates based on PFGE. [file 1746-6148-8-200-S2.pdf]

Supplementary file 2

Clustering and comparison of isolates based on PFGE

PFGE comparison, human and sheep isolates

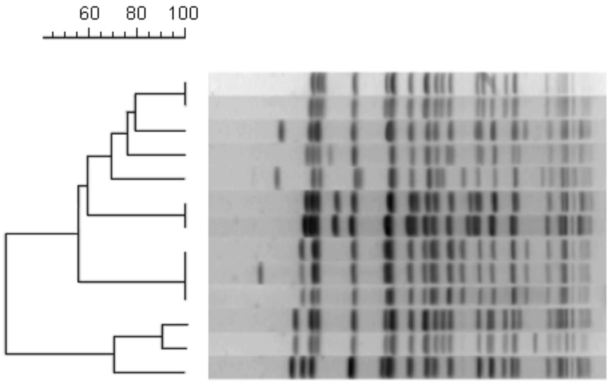

Isolate (PFGE profile)	Source	MLVA
E162/08	Human	19-8-14-3-9-7-5-10
07-BKT34471 (K)	Sheep	15-8-14-3-9-7-5-10
08-BKT5515 (La)	Sheep	5-7-14-4-6-6-NA-5
08-BKT36780 (Sa)	Sheep	5-7-12-4-6-6-7-5
08-BKT8080 (Lb)	Sheep	4-7-NA-5-3-9-5-8
08-BKT42292 (Sb1)	Sheep	5-7-13-4-9-6-7-5
08-BKT42293 (Sb2)	Sheep	5-7-13-4-9-6-7-5
08-BKT45237 (Ld1)	Sheep	14-7-11-3-4-5-9-6
08-BKT45241 (Ld2)	Sheep	14-7-11-3-4-5-9-6
E91/03	Human	14-6-6-3-4-5-5-8
08-BKT39835 (Va)	Sheep	NA-7-16-5-9-6-5-11
07-BKT35158 (Vb)	Sheep	NA-7-15-5-9-6-5-11
08-BKT45239 (Lc)	Sheep	17-7-20-4-4-7-8-5

Optimization 3%, band match tolerance 1 %. Ward clustering based on Dice similarity index

PFGE comparison, cattle and sheep isolates

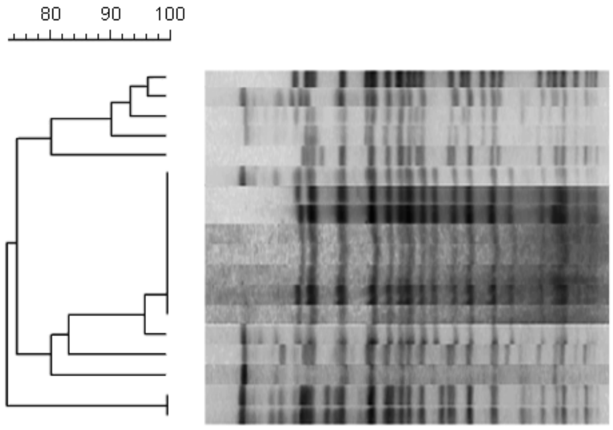

Isolate (PFGE profile)	Source	MLVA
08-BKT39835 (Va)	Sheep	NA-7-16-5-9-6-5-11
08-BKT45239 (Lc)	Sheep	17-7-20-4-4-7-8-5
07-BKT35158 (Vb)	Sheep	NA-7-15-5-9-6-5-11
07-BKT34471 (K)	Sheep	15-8-14-3-9-7-5-10
08-BKT36780 (Sa)	Sheep	5-7-12-4-6-6-7-5
08-BKT45237 (Ld1)	Sheep	14-7-11-3-4-5-9-6
T158/05	Cattle	19-7-12-3-4-5-8-7
T174/05	Cattle	16-8-11-3-4-5-6-6
T304/05	Cattle	18-8-11-4-4-5-5-6
T306/05	Cattle	17-8-11-3-4-5-7-6
T307/05	Cattle	15-7-14-5-4-5-6-6
T408/05	Cattle	19-7-11-3-4-5-7-7
T409/05	Cattle	19-7-11-3-4-5-7-7
08-BKT45241 (Ld2)	Sheep	14-7-11-3-4-5-9-6
08-BKT5515 (La)	Sheep	5-7-14-4-6-6-NA-5
08-BKT8080 (Lb)	Sheep	4-7-NA-5-3-9-5-8
08-BKT42292 (Sb1)	Sheep	5-7-13-4-9-6-7-5
08-BKT42293 (Sb2)	Sheep	5-7-13-4-9-6-7-5

Optimization 0.5%, band match tolerance 1.2 %. UPGMA clustering based on Dice similarity index
